# Surge in SARS-CoV-2 transmission in school-aged children and household contacts, England, August to October 2021

**DOI:** 10.2807/1560-7917.ES.2021.26.48.2101019

**Published:** 2021-12-02

**Authors:** Dimple Y Chudasama, Elise Tessier, Joe Flannagan, David Leeman, Harriet Webster, Alicia Demirjian, Catherine Falconer, Simon Thelwall, Meaghan Kall, Vanessa Saliba, Mary Ramsay, Gavin Dabrera, Theresa Lamagni

**Affiliations:** 1 COVID-19 Epidemiology Cell, UK Health Security Agency, London, United Kingdom; 2 Paediatric Infectious Diseases and Immunology, Evelina London Children’s Hospital, London, United Kingdom; 3 Faculty of Life Sciences & Medicine, King’s College London, London, United Kingdom; 4 Clinical & Public Health, Young People Cell, UK Health Security Agency, London, United Kingdom; 5 Surveillance Cell, UK Health Security Agency, London, United Kingdom

**Keywords:** COVID-19, SARS-CoV-2, clusters, outbreaks, children, schools, vaccination, England, United Kingdom

## Abstract

Easing of COVID-19 restrictions in England in the summer of 2021 was followed by a sharp rise in cases among school-aged children. Weekly rates of SARS-CoV-2 infection in primary and secondary school children reached 733.3 and 1,664.7/100,000 population, respectively, by week 39 2021. A surge in household clusters with school-aged index cases was noted at the start of the school term, with secondary cases predominantly in children aged 5–15 years and adults aged 30–49 years.

With over 80% of adults above the age of 50 years in receipt of two vaccine doses [1], a stepwise easing of national coronavirus disease (COVID-19) restrictions was implemented in England in the summer of 2021. The easing of restrictions included the removal of physical distancing measures, mandatory face coverings or masks from 19 July 2021 and the requirement for self-isolation of close contacts of cases who are children (below the age of 18 years 6 months) or fully vaccinated from 16 August 2021.

Schools reopened between late August and the beginning of September 2021 for the new academic year 2021/22 under new COVID-19 guidance, along with removal of the requirement for students to be placed in consistent groups (known as ‘bubbles’) to avoid mixing [2]. By 14 October 2021, over 110,000 children (1.4%) were registered as absent because of a COVID-19 diagnosis [3]. We report on the incidence of COVID-19 among the school-aged population and onward household transmission.

## Investigating SARS-CoV-2 incidence rates and clusters in school-aged children

Population rates per 100,000 of severe acute respiratory syndrome coronavirus 2 (SARS-CoV-2) infection in England were assessed from national surveillance of COVID-19 cases capturing all test methods including self-collected lateral flow device (LFD) tests reported to the United Kingdom (UK) Health Security Agency (UKHSA) up to 2 October 2021. The following school-age bands were applied: (i) pre-school, under the age of 5 years; (ii) primary school, aged 5–11 years; (iii) secondary school, aged 12–15 years and; (iv) college and sixth form, aged 16–18 years. Age-specific rates were calculated using the mid-year population estimates provided by the Office for National Statistics (ONS) [4]. Analyses of trends over time were based on date of the earliest positive specimen for both LFD and/or PCR.

## Identification of household clusters

Cases’ residential addresses were matched to reference databases to obtain the unique property reference number (UPRN) and type of residence [5]. Analyses were restricted to individuals identified as residents in a private dwelling. The data from UPRN were used to identify clusters, defined as two or more cases resident at the same dwelling within a rolling 14-day window.

Analyses of household clusters were restricted to those where a single case was identified as the index i.e. no further cases were diagnosed within the first 2 days of the index case’s detection. Assessment of secondary cases was restricted to the next sequential case diagnosed within a household after the index case. Where more than one secondary case was diagnosed on the same day, all were included in analyses.

## Rising rates of SARS-CoV-2 infection in children over the summer

**Figure 1:**
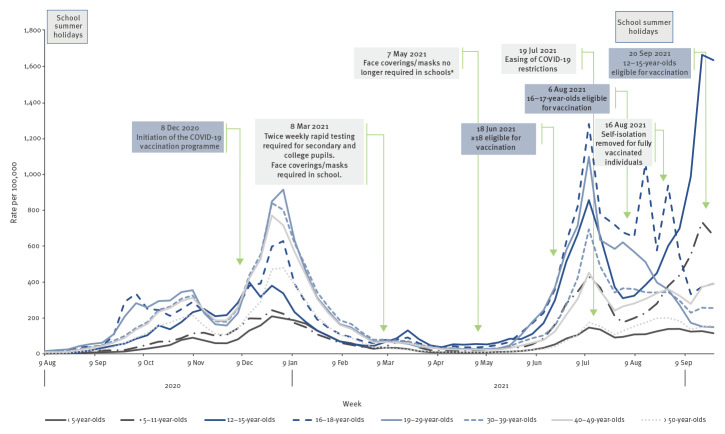
Rate of COVID-19 cases per 100,000 population by age groups, England, 9 August 2020–2 October 2021

COVID-19: coronavirus disease; SARS-CoV-2: severe acute respiratory syndrome coronavirus 2.

^a^ Face coverings or masks can be reintroduced in outbreak control situations in line with the contingency framework.

Following the declines in rates of COVID-19 since peak activity in mid-July 2021 (Figure 1), rates rose steeply among children aged 5–15 years from the beginning of August 2021, with the rise most pronounced in those of secondary school age.

Rates peaked at 733.3 and 1,664.7 per 100,000 population in primary and secondary school-aged populations in week 38 2021 (from 19 September to 25 September), respectively, whereas a sharp decline for 16–18-year-olds was observed reaching 373.0 per 100,000 population during the same week. Vaccination rollout began on 18 June 2021 for those aged 18 years and older and on 6 August 2021 for 16–17-year-olds. Vaccine coverage for 16–17-year-olds reached 55.4% and 15.6% for the first and second dose, respectively, and 9.0% and 0.2%, respectively in 12–15-year-olds, as at 7 October [6]. The majority of cases aged 5–18-years, 74.9% (n = 234,730/313,218), reported between 1 September and 2 October were diagnosed by PCR. Compared with the period before school opening (1–21 August 2021), a sequential increase in case detection through LFD testing was seen during school reopening (from 22 August to 10 September 2021), and immediately after (from 11 September to 2 October 2021), from 24.5% to 32.6%, in line with the schools asymptomatic testing programme for 12–15-year-olds (Table). The proportion of cases reported as being symptomatic also fell slightly during this period, from 51.3% to 49.9%.

**Table:** COVID-19 cases and household cluster index cases by detection period and SARS-CoV-2 test methods used, children aged 12–15 years, England, 1 August–2 October 2021 (n = 195,103 cases and n = 24,683 cluster index cases)^a^

Before return to school (1–21 Aug 2021, week 31−week 33)					Return to school (22 Aug–10 Sep 2021, week 34−week 36)				After return to school (11 Sep–2 Oct 2021, week 37−week 39)			
**All diagnosed cases**												
**Tests**	**Total cases**	**% of all tests**	**Number of symptomatic cases**	**% of test method^b^**	**Total cases**	**% of all tests**	**Number of symptomatic cases**	**% of test method^b^**	**Total cases**	**% of all tests**	**Number of symptomatic cases**	**% of test method^b^**
LFD only	1,088	4.4	1	0.1	1,988	4.8	11	0.6	4,353	3.9	11	0.3
LFD with PCR	4,914	20.1	1	0.0	9,988	24.1	27	0.3	31,960	28.7	21	0.1
all LFD	6,002	24.5	2	0.0	11,976	29.0	38	0.3	36,313	32.6	32	0.1
PCR only	18,468	75.5	12,547	67.9	29,390	71.0	19,447	66.2	75,127	67.4	55,495	73.9
All tests	24,470	100.0	12,549	51.3	41,366	100.0	19,485	47.1	111,440	100.0	55,559	49.9
Household cluster index cases												
LFD only	106	4.2	0	0.0	184	3.9	1	0.5	471	2.9	1	0.2
LFD with PCR	803	32.0	0	0.0	1,605	34.3	7	0.4	6,421	39.4	4	0.1
all LFD	909	36.3	0	0.0	1,789	38.2	8	0.4	6,892	42.3	5	0.1
PCR only	1,598	63.7	1,241	77.7	2,894	61.8	2,219	76.7	9,395	57.7	7,522	80.1
All tests	2,507	100.0	1,241	49.5	4,683	100.0	2,227	47.6	16,287	100.0	7,527	46.2

LFD: lateral flow device; PCR: polymerase chain reaction; SARS-CoV-2: severe acute respiratory syndrome coronavirus 2.

^a^ 17,827 cases (1,206 index cluster cases) without information on symptomatic status were excluded.

^b^ Presented as row percentages.

## Increase in household clusters with school-aged children as the index case

**Figure 2:**
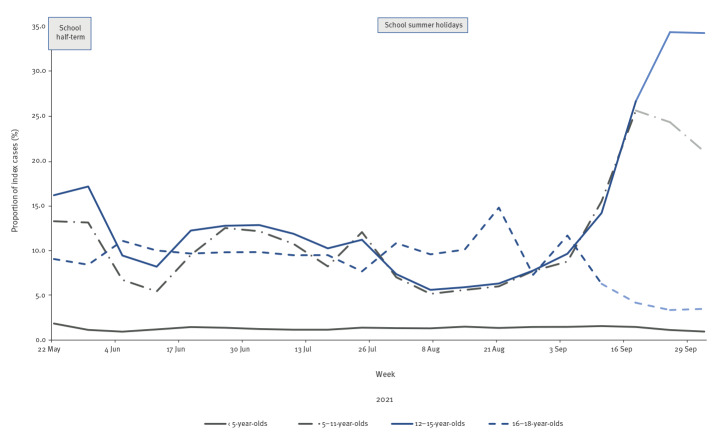
Proportion of index cases aged 18 years and younger among total household clusters by week, England, 22 May–2 October 2021^a^

^a^ Data for the last 2 weeks shown are subject to change as further outbreaks develop and are identified.

The proportion of household clusters where a child (aged 5–15 years) was identified as the index case remained broadly similar over the summer of 2021 (Figure 2). However, a sharp increase was noted from week 35 2021 (from 29 August to 4 September), peaking in week 38 2021 (from 19 September to 25 September); this was more pronounced for index cases aged 12–15 years compared with index cases aged 5–11 years, accounting for 34.3% (n = 7,739/22,538) and 24.3% (n = 5,476/22,538) of all household clusters respectively during that week (Figure 2). The same period saw a gradual decline in 16–18-year-olds identified as being the index case of a cluster. Compared with all cases, a higher proportion of household cluster index cases (aged 12–15 years) were diagnosed by LFD testing (36.3% vs 24.5% before school return; 38.2% vs 29.0% upon returning to school; 42.3% vs 32.6% after returning to school).

**Figure 3:**
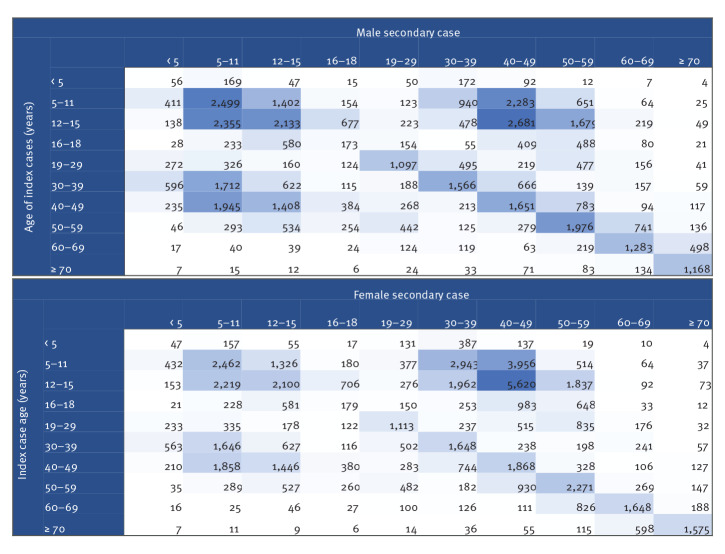
Age of index cases and the secondary case by sex in household clusters, England, 1 September–4 October 2021^a^

^a^ Heatmap grading is per totals and thus independently calculated per table, therefore direct comparisons of shading cannot be made.

Within household clusters with index cases who were primary and secondary school-aged children, counts of secondary cases were higher among school-aged children (particularly within males) and adults aged 30–59 years, particularly among 40–49-year-olds (Figure 3). Notably, adult secondary cases linked to school-aged index cases were more likely to be female than male, particularly among 30–49-year-olds (9,576 vs 4,963). Transmission from adults aged 40–49 years to children aged 5–15 years was also apparent. The median interval between positive specimens in school-aged index and secondary cases was 3 days (interquartile range: 2–5) for clusters detected from 1 September to 2 October 2021.

### Ethical statement

All data were collected within statutory approvals granted to UKHSA for infectious disease surveillance and control. Information was held securely and in accordance with the Data Protection Act 2018 and Caldicott principles.

## Discussion

Following the sharp spike in COVID-19 incidence in July 2021 which coincided with the 11 July European Football Championship (EURO 2020) final, rates fell across all age groups. Although rates fell in 16–18-year-olds over the course of August 2021, spikes that were potentially linked to attendance at summer festivals were seen. In contrast to adults, rates in children aged 5–15 years subsequently began to increase in August following the easing of national COVID-19 restrictions on 19 July 2021. While high uptake of the COVID-19 vaccine in adults was achieved by this point [1], the vaccine rollout to 12–15-year-olds was not launched until 20 September 2021 [7]. Further accelerations in rates for this age group were seen following the reopening of schools at the end of August and beginning of September 2021. Weekly test positivity data reported by ONS and UKHSA showed a marked rise in positivity rate among secondary school-aged children over this period [8,9]. A similar rise was seen at the start of the previous (2020/21) academic year in England [10], however, the magnitude of the rise has been more pronounced this year. The differences could in part be attributable to increased transmissibility of the SARS-CoV-2 Delta (Phylogenetic Assignment of Named Global Outbreak (Pango) lineage designation B.1.617.2 and AY.* sublineages) compared with the Alpha variant [11,12]. Research to date has suggested limited transmission of SARS-CoV-2 in school settings in the UK, with infections in children and COVID-19 outbreaks in schools reflecting levels of wider community transmission [11,13].

Our findings illustrate the wider impact of cases in children, with an increase in household clusters observed in both primary and secondary school-aged index cases. While we cannot determine definitively that subsequent cases within the household represent transmission from index cases, the risk from cohabitation makes this likely [14-17]. The rise in clusters seeded by secondary school-aged children may reflect in part the systematic asymptomatic testing of this age group at the start of the school term, leading to increased testing among household contacts. This would not, however, account for the rise in clusters with primary school-aged index cases. The large number of children who were secondary cases in these clusters were likely to be siblings of the school-aged index case, raising the prospect for further transmission in other schools or year groups since the requirement for household contacts to self-isolate has been suspended [18]. Furthermore, secondary household cases were seen across all age groups including elderly people; while most adults are afforded protection from vaccination, this is not universally or uniformly the case [19].

Countries worldwide continue to grapple with balancing the needs of children to be in school while controlling spread of COVID-19 [20]. Keeping schools open for on-site learning is essential for the wellbeing of children, particularly for those with challenging home environments. While the immediate health risks of SARS-CoV-2 infection to children are low, the harms of disruption to education and general wellbeing can be substantial [21]; school absences for suspected and confirmed COVID-19 cases rose sharply in the first few weeks of the new term, sparking concern about the impact on both education and delivery of the school-based vaccine programme [22]. Additional protective measures may be of benefit; a study from the United States found a lower incidence of COVID-19 in counties where schools required the use of face coverings or masks in combination with other prevention strategies [23].

### Conclusion

Easing of national COVID-19 restrictions over the summer of 2021, shortly after a period of high incidence and ahead of the initiation of the childhood vaccination programme, resulted in a sharp rise in cases of COVID-19 among children. While further increases in rates among children were seen, in due course we anticipate a positive impact following the recent rollout of the vaccination programme in 12–15-year-olds on protecting children and their education, contingent on uptake.

## References

[1] United Kingdom Government. (GOV.UK). Weekly national Influenza and COVID-19 surveillance report. Week 29 report (up to week 28 data). London: GOV.UK; 2021. Available from: https://assets.publishing.service.gov.uk/government/uploads/system/uploads/attachment_data/file/1005056/Weekly_Flu_and_COVID-19_report_w29.pdf

[2] United Kingdom Government. (GOV.UK). Department for Education. Schools COVID-19 operational guidance. London: GOV.UK; 2021. Available from: https://www.gov.uk/government/publications/actions-for-schools-during-the-coronavirus-outbreak/schools-covid-19-operational-guidance

[3] United Kingdom Government. (GOV.UK). Table 1B - Daily attendance in education settings during the COVID-19 outbreak (excludes schools holiday dates). London: GOV.UK; 2021. Available from: https://explore-education-statistics.service.gov.uk/data-catalogue/attendance-in-education-and-early-years-settings-during-the-coronavirus-covid-19-outbreak/2021-week-42

[4] Office For National Statistics (ONS). ONS mid-year population 2020. Newport: ONS; 2020. Available from: https://www.ons.gov.uk/peoplepopulationandcommunity/populationandmigration/populationestimates/datasets/populationestimatesforukenglandandwalesscotlandandnorthernireland

[5] Chudasama DY, Milbourn H, Nsonwu O, Senyah F, Florence I, Cook B, et al. Penetration and impact of COVID-19 in long term care facilities in England: population surveillance study. Int J Epidemiol. 2021;dyab176. 10.1093/ije/dyab176 34999883

[6] UK Health Security Agency (UKHSA). Weekly national Influenza and COVID-19 surveillance report Week 40 report (up to week 39 data) 2021. London: UKHSA; 2021. Available from: https://assets.publishing.service.gov.uk/government/uploads/system/uploads/attachment_data/file/1023910/Weekly_Flu_and_COVID-19_report_w40_v2.pdf

[7] NHS. NHS rolls out COVID-19 jab to children aged 12 to 15. London: NHS; 2021. Available from: https://www.england.nhs.uk/2021/09/nhs-rolls-out-covid-19-jab-to-children-aged-12-to-15

[8] UK Health Security Agency (UKHSA). Weekly Influenza and COVID 19 Surveillance graphs. London: UKHSA; 2021. Available from: https://assets.publishing.service.gov.uk/government/uploads/system/uploads/attachment_data/file/1025454/Weekly_COVID-19_and_Influenza_Surveillance_Graphs_w41_v2.pdf

[9] Office For National Statistics (ONS). Coronavirus (COVID-19) infection survey, UK. Newport: ONS; 2021. Available from: https://www.ons.gov.uk/peoplepopulationandcommunity/healthandsocialcare/conditionsanddiseases/bulletins/coronaviruscovid19infectionsurveypilot/8october2021

[10] Aiano F Mensah AA McOwat K Obi C Vusirikala A Powell AA COVID-19 outbreaks following full reopening of primary and secondary schools in England: Cross-sectional national surveillance, November 2020. Lancet Reg Health Eur. 2021;6:100120. 10.1016/j.lanepe.2021.100120 34278370 PMC8276523

[11] Young BC Eyre DW Kendrick S White C Smith S Beveridge G Daily testing for contacts of individuals with SARS-CoV-2 infection and attendance and SARS-CoV-2 transmission in English secondary schools and colleges: an open-label, cluster-randomised trial. Lancet. 2021;398(10307):1217-29. 10.1016/S0140-6736(21)01908-5 34534517 PMC8439620

[12] Allen H Vusirikala A Flannagan J Twohig KA Zaidi A Chudasama D Household transmission of COVID-19 cases associated with SARS-CoV-2 delta variant (B.1.617.2): national case-control study. Lancet Reg Health Eur. 2021;00:100252. 10.1016/j.lanepe.2021.100252 34729548 PMC8552812

[13] Ladhani SN Baawuah F Beckmann J Okike IO Ahmad S Garstang J SARS-CoV-2 infection and transmission in primary schools in England in June-December, 2020 (sKIDs): an active, prospective surveillance study. Lancet Child Adolesc Health. 2021;5(6):417-27. 10.1016/S2352-4642(21)00061-4 33740430 PMC9764982

[14] D’Onofrio LE Jr Buono FD Cooper MAR . Cohabitation COVID-19 transmission rates in a United States suburban community: A retrospective study of familial infections. Public Health. 2021;192(30-32):30-2. 10.1016/j.puhe.2021.01.003 33611168 PMC7816609

[15] Miller E Waight PA Andrews NJ McOwat K Brown KE Höschler K Transmission of SARS-CoV-2 in the household setting: A prospective cohort study in children and adults in England. J Infect. 2021;83(4):483-9. 10.1016/j.jinf.2021.07.037 34348116 PMC8325949

[16] Cordery R, Reeves L, Zhou J, Rowan A, Watber P, Rosadas C, et al. Transmission of SARS-CoV-2 by children in schools and households: a prospective cohort and environmental sampling study in London. medRxiv. 2021. Preprint. https://doi.org/10.1101/2021.03.08.21252839 10.1016/S2666-5247(22)00124-0 PMC9401977 36029775

[17] Hall JA Harris RJ Zaidi A Woodhall SC Dabrera G Dunbar JK . HOSTED-England’s Household Transmission Evaluation Dataset: preliminary findings from a novel passive surveillance system of COVID-19. Int J Epidemiol. 2021;50(3):743-52. 10.1093/ije/dyab057 33837417 PMC8083300

[18] United Kingdom Government. (GOV.UK). Guidance for contacts of people with confirmed coronavirus (COVID-19) infection who do not live with the person. London: GOV.UK; 2021. Available from: https://www.gov.uk/government/publications/guidance-for-contacts-of-people-with-possible-or-confirmed-coronavirus-covid-19-infection-who-do-not-live-with-the-person

[19] Tessier E, Rai Y, Clarke E, Lakhani A, Tsang C, Makwana A, et al. Characteristics associated with COVID-19 vaccine uptake among adults in England (08 December–17 May 2021). medRxiv. 2021. Preprint. https://doi.org/10.1101/2021.08.27.21262422

[20] Murdoch children's research institute (MCRI). Research Brief COVID-19 in Early Childhood Education and Care and Schools. Melbourne: MCRI; 2021. Available from: https://www.mcri.edu.au/sites/default/files/media/documents/covid-19_in_early_childhood_education_and_care_and_schools.pdf

[21] Viner R, Russell S, Saulle R, Croker H, Stansfeld C, Packer J, et al. Impacts of school closures on physical and mental health of children and young people: a systematic review. medRxiv. 2021. Preprint. https://doi.org/10.1101/2021.02.10.21251526

[22] United Kingdom Government. (GOV.UK). Attendance in education and early years settings during the coronavirus (COVID-19) outbreak. London: GOV.UK; 2021. Available from: https://explore-education-statistics.service.gov.uk/find-statistics/attendance-in-education-and-early-years-settings-during-the-coronavirus-covid-19-outbreak

[23] Budzyn SEPM Panaggio MJ Parks SE Papazian M Magid J Eng M Pediatric COVID-19 cases in counties with and without school mask requirements - United States, July 1-September 4, 2021. MMWR Morb Mortal Wkly Rep. 2021;70(39):1377-8. 10.15585/mmwr.mm7039e3 34591829 PMC8486393

